# Color Duplex Assessment of 4th and 5th Internal Mammary Artery Perforators: The Pedicles of the Medially Based Lower Pole Breast Flaps

**Published:** 2012-01-23

**Authors:** Kareem Abdel-Monem, Ahmed Elshahat, Sherif Abou-Gamrah, Hossam Eldin Abol-Atta, Reda Abd Eltawab, Karim Massoud

**Affiliations:** ^a^Plastic Surgery Department; ^b^Radiodiagnosis Department; ^c^General Surgery Department, Faculty of Medicine, Ain Shams University, Abbassia, Cairo, Egypt

## Abstract

**Objective:** Reconstruction of a breast after mastectomy using the contralateral lower pole breast flap is an appealing procedure because it uses the tissues that were going to be excised during reduction of the sound breast to achieve symmetry. Literature mentioned that these flaps are supplied by the lower internal mammary artery perforators (IMAPs) with no further details. The aim of this study was to determine the site, size, and number of the 4th and 5th IMAPs by using preoperative color Duplex ultrasound and intraoperative exploration. **Method:** Twenty breasts in 10 patients who presented for reduction mammoplasty were included in this study. Preoperative color duplex was used to determine IMAPs in the 4th and 5th intercostal spaces. These perforators were localized intraoperatively. Intravenous fluorescein injection was used to determine the perfusion of the lower pole breast flap on the basis of these perforators. **Results:** Statistically, the 4th IMAPs diameters were significantly larger than the 5th IMAPs diameters (*P* < .05). The lower pole breast flap was perfused through these perforators. **Conclusion:** Color Duplex ultrasound is an accurate tool to preoperatively determine the 4th and 5th IMAPs.

Breast reconstruction following mastectomy is a feasible option to help in restoring the body's shape and image of the afflicted women. There are multiple surgical procedures for the reconstruction of the female breast, including the use of a mammary prosthesis (implant) or various autologous tissues.

Theoretically, regarding autologous reconstruction, the lowest donor site morbidity could be achieved by using tissue that would be otherwise rejected during an operation necessary for a different reason. Adoption of this principle was the cause behind the introduction of the transverse rectus abdominis myocutaneous flap for breast reconstruction.^1^^,^^2^ Likewise, in properly selected postmastectomy patients in whom the remaining breast has to be reduced anyway because of hypertrophy, the resected part of such a reduction mammoplasty can be used as a tissue source for the other breast reconstruction.

Many works (both anatomical and review studies) concerning chest wall and breast arterial supply had appeared since the turn of the twentieth century. One of the early and technically acceptable researches was Salmon injection study in 1939,^3^ in which he concluded that the arteries of the breast take their origin from 3 systems: axillary, internal mammary, and intercostal arteries. Salmon^3^ stated that the first 2 systems were the major sources of mammary blood while the aortic contribution was minor.

More acknowledged, and hence more commonly cited studies in plastic surgery literature describing the anterior chest wall, including the breast, vascular territories (referred to as angiosomes) were published later.^4^^,^^5^

The internal mammary artery perforators (IMAPs) provide the vascular basis for many cutaneous (glandulocutaneous in women) flaps in the region of anterior chest wall, with variable reconstructive applications for the breast,^6^^-^12 the anterior chest wall, and head and neck regions.^13^^-^16

The articles that published the use of flaps based on the lower IMAPs for the chest wall and the contralateral breast reconstruction^6^^-^12^,^^17^ presented no precise data regarding the exact name, size, location, and the number of perforators that were included in these flap pedicles.

Whereas Schoeller and colleagues,^12^ in 2001, considered the 4th and 5th IMAPs to be the main blood supply to the lower part of the breast, other authors^4^^,^^15^^,^^18^^,^^19^ considered that the 4th IMAPs are the main supply to the lower part of the breast while the 5th ones are responsible for the tissues caudal to the inframammary folds.

Doppler ultrasonography is a highly valuable and practical contrivance that plastic surgeons use for preoperative mapping of perforating vessels throughout the cutaneous territory of a flap, aiming at improving their surgical strategies so that the operative procedures can proceed in a faster and safer way. Several early introduced studies using color Duplex provided useful information related to the location, caliber, and flow patterns of the perforators in the planning of the transverse rectus abdominis myocutaneous flap.^20^^,^^21^

In 1998, Blondeel and coworkers^22^ reviewed their experience in using preoperative sonographical assessment. They appreciated and acknowledged the usefulness of the color Duplex, in terms of providing preoperatively the necessary information on vascular anatomy of the used flaps.

Schoeller et al^12^ and Dian et al^17^ conducted Doppler sonographic studies for the IMAPs before elevating flaps based on them. They detected the perforators but gave no details regarding site, size, or exact location.

In 2010, Schmidt and associates^19^ investigated the vascular basis of the IMAP flap and described the location and diameter of the individual IMAPs, but through a cadaveric injection study. However, they advised conducting a preoperative Doppler sonography on the IMAPs in clinical situation.

The aim of this study was to determine the site, size, and number of the IMAPs occupying the 4th and 5th intercostals spaces in patients presented for reduction mammoplasty, using color Duplex ultrasound and intraoperative exploration. In addition, this study ensures the adequacy of these perforators to supply the lower pole breast flaps.

## PATIENTS AND METHODS

This study was conducted on 10 female patients with bilateral huge breasts who presented for reduction mammoplasty. A signed written informed consent was obtained from each patient regarding her agreement on participation in this study. A preoperative sonographic assessment of the internal mammary artery perforating branches in the 4th and 5th interspaces was performed, followed by intraoperative double check of the Duplex-obtained data.

### Sonographic technique

A color Duplex scanner (LOGIQ 7 PRO: General Electric Yokogawa Medical Systems Ltd, Tokyo, Japan) was used to preoperatively visualize the perforation site (distance from the lateral sternal border), the diameter, and identify the number of detectable IMAPs (if any) in each of the 2 intercostal spaces mentioned earlier. To avoid potential errors caused by different interpretations, as ultrasound explication is operator dependent, this interpretation was done by a single radiologist (Abou-Gamrah), experienced in Duplex assessment of small vessels.

**Figure 1:**
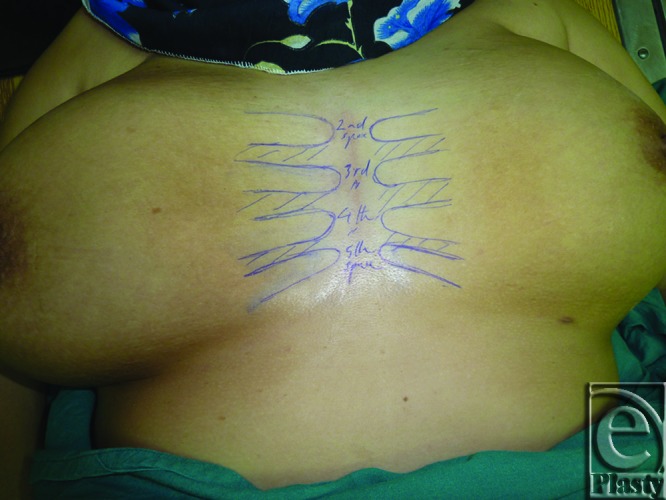
Anterior chest wall of a woman in the supine position showing pre-Duplex marking of the 2nd through the 5th intercostal spaces.

At first, each patient was placed in the supine position, and then the intercostal spaces from the 2nd–5th were marked on both sides of the chest (Fig 1). Parasternal regions at the 4th and 5th spaces were bilaterally scanned using B-mode ultrasonography with a linear probe frequency of 12 MHz after adjusting the B-mode gain to clearly visualize the deep fascia, and then a color Duplex with pulsed Doppler wave was used to detect the perforators after adjusting the following parameters: pulse repetition frequency at low setting level to detect low velocities; color gain to avoid over- or underestimation of the perforator's diameter; the Doppler angle to be less than 60°; and the sample volume of the Doppler beam.

**Figure 2:**
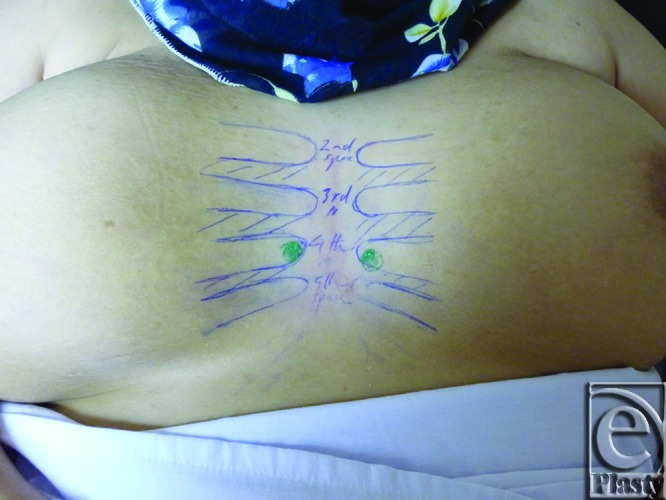
Anterior chest wall of the same female in Figure 1 showing post-Duplex labeling of the visualized perforator sites in order to measure how far laterally they are from the sternum. No markings are seen in the 5th space in this photo because the 5th perforators were absent in this patient.

**Figure 3:**
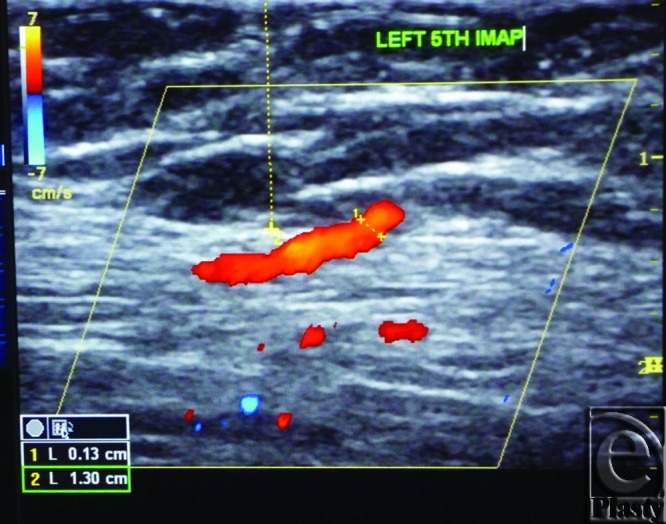
Color Duplex photo shows the diameter and depth of a certain visualized 5th internal mammary artery perforator from the skin surface. The point of measurement was at the perforation point.

**Figure 4:**
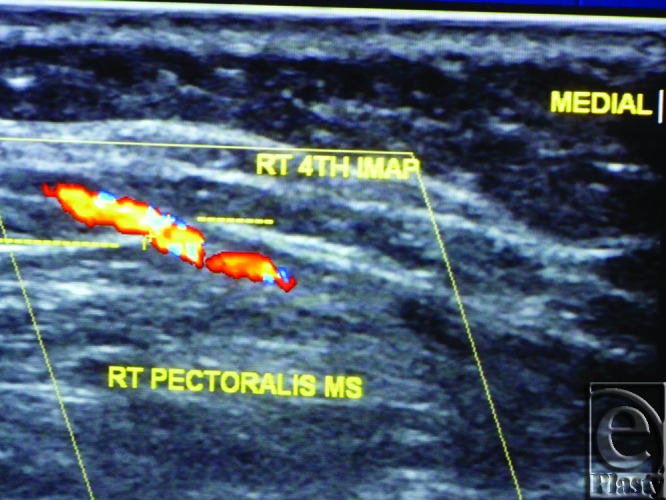
Color Duplex photo shows a 4th internal mammary artery perforator while perforating the superior surface of the pectoralis major muscle (the dashed line).

**Figure 5:**
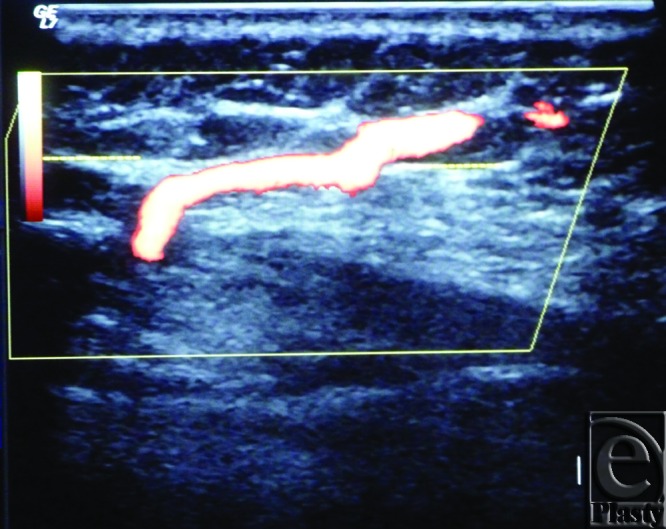
Color Duplex photo shows an intramuscular course (below the dashed line) of an internal mammary artery perforator before emerging through the pectoralis surface (the dashed line itself) to the overlying breast tissue.

**Figure 6:**
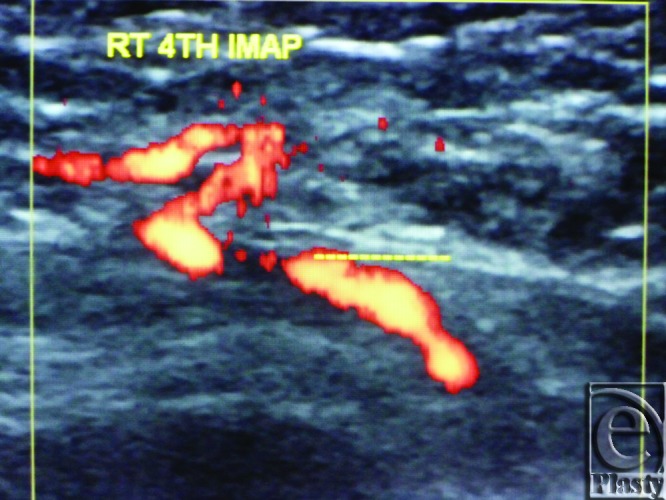
Color Duplex photo shows the highly tortuous course (above the dashed line) of one internal mammary artery perforator while traversing through the breast tissue fat just after perforating the pectoralis major (the dashed line).

**Figure 7:**
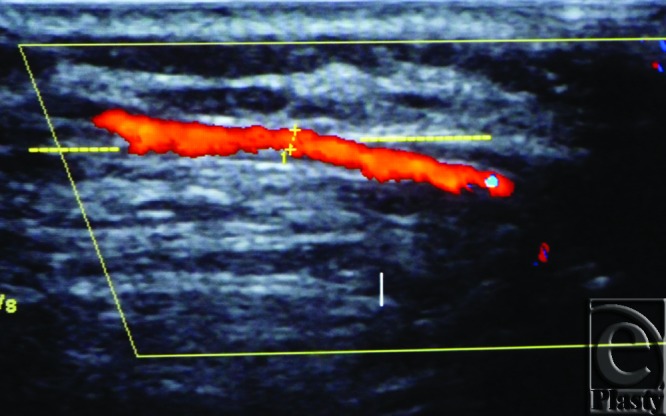
Color Duplex photo shows another potential variation in the trajectory of an internal mammary artery perforator that coursed tangential to the fascia overlying the pectoralis (dashed line) for a distance.

**Figure 8:**
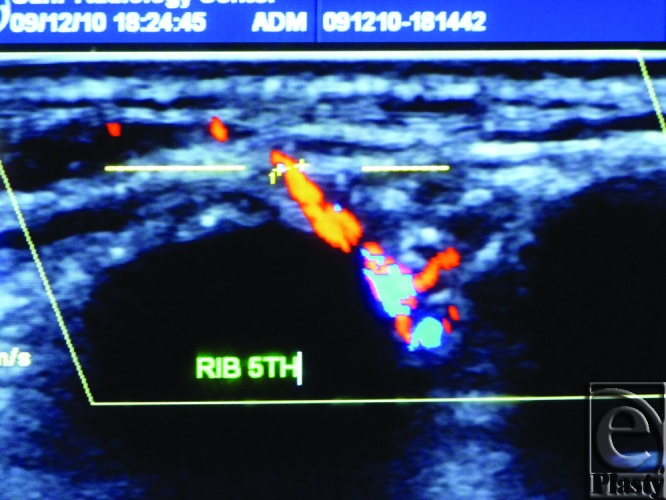
Color Duplex photo shows a 5th internal mammary artery perforator seen while passing between the 4th and 5th ribs (the two consecutive jet-black areas) before perforating the overlying pectoralis major (the dashed line).

The site of the detectable perforator(s) was projected by a colored marker on the patient's skin so as to estimate the label remoteness (representing the perforation site) from the sternum (Fig 2), and the number of encountered perforators parasternally in each investigated space was counted as well. The inner diameter of each perforator was measured (Fig 3). Figures 4 to 8 show color duplex photos for perforators penetrating the fascia to reach the dermoglandular target.

Data were recorded for analysis, and documentation was done by ultrasound photos. The findings collated were compared with anatomic data obtained later intraoperatively.

### Data management and analysis

The collected data were introduced to a personal computer using statistical package for social science (SPSS 15.0.1 for windows; SPSS Inc, Chicago, Ill, 2001). Paired *t* test was used to statistically compare 2 values. A *P* < .05 was assumed as significant.

### Surgical technique

**Figure 9:**
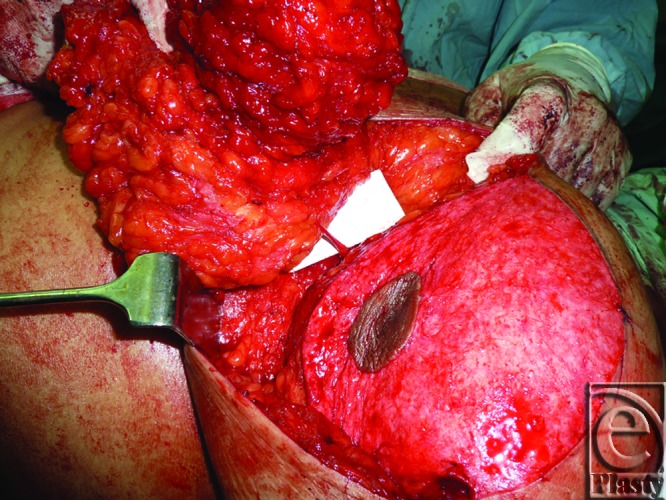
Intraoperative view of a left-side breast during reduction mammoplasty using the superior pedicle technique. Skeletonized 4th internal mammary artery perforator (on the white background) is shown while entering to the going-to-be excised lower breast tissue just before intravenous fluorescein injection.

**Figure 10:**
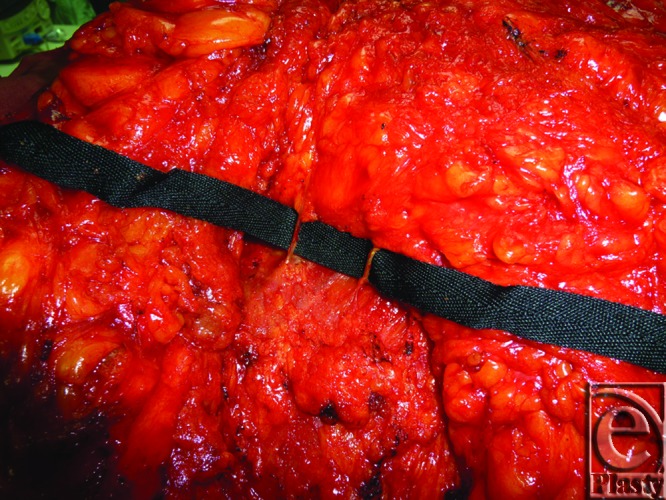
Intraoperative view of a right-side breast during reduction mammoplasty shows the 4th and 5th internal mammary artery perforators entering the still medially attached lower breast tissue before completion of excision and before intravenous fluorescein injection.

**Figure 11:**
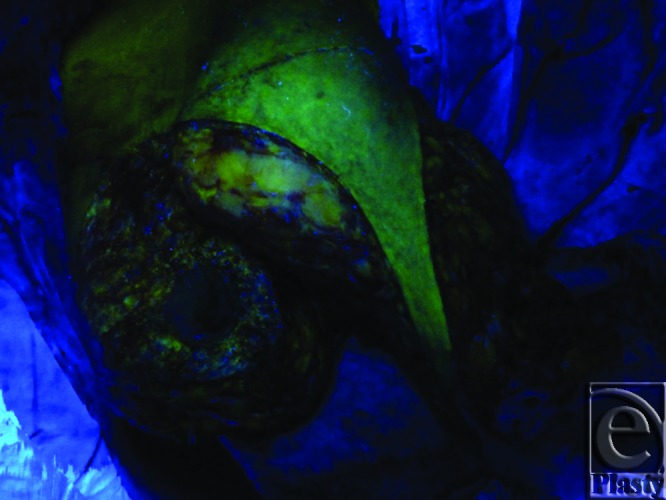
Intraoperative view of a breast during reduction mammoplasty shows yellow-green fluorescence of a circumferentially isolated, yet medially attached inferior breast pole tissue mass 20 minutes after intravenous fluorescein injection indicating real-time tissue perfusion. This photo was taken under ultraviolet light while the room is darkened.

**Figure 12:**
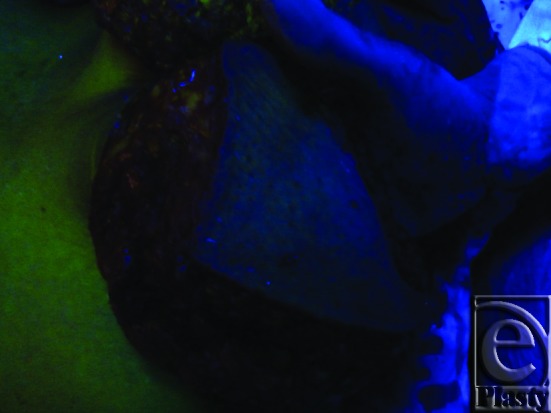
Intraoperative view of a breast during reduction mammoplasty shows nonfluorescent (dark blue) medially attached lower breast tissue. This reflects nonvascularization at the time of dye injection (due to accidental internal mammary artery perforator injury). Note that the surrounding perfused tissues show yellow-green fluorescence. This photo was taken under ultraviolet light while the room is darkened.

A superior-pedicle-based reduction mammoplasty was performed for all patients. The excess inferior lipoglandulocutaneous part of each breast was dissected to its medial border to visualize any potential perforator(s) emerging from the 4th and 5th interspaces parasternally, and entering this tissue mass (Figs 9 and 10). After perforator(s) identification, the patient was intravenously injected with 20 mg/kg fluorescein dye (fluorescein sodium) under supervision of the anesthesia team, and after 15 to 20 minutes a Wood's lamp was used, with the room light off, to evaluate the fluorescence of this inferior part of the breast going to be excised, and thus its real-time perfusion (Figs 11 and 12). Before dye injection, all the patients were tested for sensitivity to the dye by injecting 0.05 mL intradermally. Then the identified perforator(s) was divided, and excision of the excess inferior breast tissue was completed.

## RESULTS

Ten women, aged between 28 and 42 years (mean 33.2 ± 4.1 SD), with bilateral gigantomastia needing reduction mammoplasty were included in this series. Both breasts of all patients (n = 20) were examined preoperatively with color Duplex seeking to visualize and identify the aforementioned parameters pertaining to the 4th and 5th IMAPs (Table 1).

As the mean distance measured from suprasternal notch to nipple-areola complex (NAC) on the right (n = 10) equaled to that on the left (n = 10) breasts, being 39.2 cm (range, from 35 to 48m on the right side, and from 35 to 47 cm on the left side), so the NAC remoteness from suprasternal notch was not significantly different between both breast sides (Tables 1 and 2).

The mean diameter measured by Duplex of the 20 perforators on the right chest wall was 1.06 ± 0.38 mm (ranged from 0.6 to 2.1 mm), and their average location lateral to the right lateral sternal edge was 1.22 ± 0.49 cm (ranging between 0.5 and 2.5 cm) (Table 3).

The left-side perforators were visualized by Duplex at a mean distance of 1.05 ± 0.6 cm (ranging between 0.5 and 2.5 cm) lateral to the left lateral sternal border, and they had a mean diameter of 1.14 ± 0.4 mm (Table 3).

Statistically, it was found that the right-side assessed group of perforators was neither significantly larger (*P* = .509) nor significantly more away from the sternum (*P* = .368) than their counterparts on the opposite chest side (Table 3).

When it came to comparing the 4th space perforators collectively of both sides (*n* = 20) with the 5th space IMAPs (*n* = 17) regarding their number, distribution, and diameter, we found that in all cases, a single IMAP only was sonographically visualized in each 4th intercostal space, but the situation was different with the IMAPs in the 5th spaces (Table 1). While, on one hand, there were 2 detectable IMAPs in the 5th space bilaterally in a single same patient, no IMAP could be identified by Duplex in the 5th intercostal spaces of some other patients (n = 5) (Table 1).

Despite this discrepancy encountered in distribution between the 2 IMAP groups, statistically, the 4th IMAPs were not significantly more numerous (*P* = .765) than the 5th ones. But, contrariwise, the both sides of 4th IMAPs were significantly larger in diameter (*P* < .05) than their counterparts in the 5th spaces bilaterally (mean diameters were 1.23 ± 0.34 mm, and 1.14 ± 0.4 mm, respectively, for both perforator groups) (Table 4).

With respect to the mean distance from the sternal margin, it was not significantly different (*P* = .546) between the aforementioned perforator groups (Table 4).

Intraoperatively, further meticulous lateral-to-medial parasternal dissection for the going-to-be excised lower glandulocutaneous breast tissue—in order to check the validity (as to the number and approximate location) of the preoperatively sonography-gained IMAP-related data (Figs 9 and 10)—showed concordant results with those obtained before surgery.

The subsequent intravenous injection of fluorescein dye, before completing the division of the still parasternally attached medial flap border, in order to evaluate the tissue flap viability demonstrated an IMAP-dependent perfusion of this raised glandulocutaneous tissue part in all cases but one (*n* = 19) (Figs 11 and 12).

## DISCUSSION

A cutaneous perforating branch, namely IMAP, is given off by the internal mammary artery in each of the first 5 to 6 intercostal spaces laterodorsal to the lateral border of the sternum. The IMAPs pierce the medial fibers of pectoralis major and finally penetrate the overlying fascia. Then these vessels traverse superficially in subcutaneous tissue in a lateral-to-laterocaudal direction to supply the skin of the ventromedial thorax and the medial two-thirds of the breast in a sequential order, with an overlap of supplied skin zones between consecutive perforators. In women, the 3rd and 4th space perforators tend to be large as they contribute to the arterial supply of the breast.^4^^,^^15^^,^^19^^,^^23^

A breast MRI study^24^ that was conducted on 26 patients and published in late 2010 found that the main source vessels to the NAC were the IMAPs (the medial source vessels). They constituted 73% of source vessels to the NAC on the right sides of the examined patients and 65% on the left sides.^24^

With regard to the 4th and 5th IMAPs specifically, the 4th IMAP has been proven through previous injection cadaveric studies to contribute to the blood supply of the areola, in addition to its typically supplied skin zone inferior to the areola cranially to the submammary fold. The skin of the proximal abdominal wall caudally to the inframammary fold is nourished by the 5th IMAP.^4^^,^^15^^,^^18^^,^^19^

In this current study, elevation of lower pole dermoglandular breast tissue on the 4th IMAP alone (in patients with undetectable 5th IMAP) did not compromise the vascularity. This proves that the 4th IMAP nourishes the whole lower pole of the breast.

Despite that raising flaps based on the lower IMAPs (other than the first 3 perforators), to be used in chest wall defect or contralateral breast reconstructions, has been reported in some literatures.^6^^-^12^,^^17^ They all presented no precise data regarding the exact name, size, location, and the number of perforators that were included in these flap pedicles.

In 2010, Schmidt and associates^19^ investigated the vascular basis of the IMAP flap and described the location and diameter of the individual IMAPs, but through a cadaveric injection study. However, they advised conducting a preoperative Doppler sonography on the IMAPs in clinical situation.

The preoperative color Duplex scan results were affirmed intraoperatively by identifying the preoperatively sonography-detected IMAPs, and then visualizing and evaluating the perfusion of the raised breast tissue flaps in real time through intravenous fluorescein injection. There were no serious complications from the use of the dye in our series except for transient nausea in some patients.

The intravenous fluorescein test is a simple, reliable, and safe (nontoxic) measure of tissue perfusion that has been used successfully to predict skin flap viability at the time of operation. When given intravenously, the fluorescein rapidly diffuses from the intravascular to the interstitial (extracellular) space where it can be seen with conventional ultraviolet light (Wood's lamp) in a darkened room. Fluorescein absorbs light in the ultraviolet range and emits light in the visible range, with a yellow-green glow. Thus, after intravenous administration of fluorescein, all vascularized tissue appears under ultraviolet light as bright yellow-green. Areas without blood supply appear dark blue.^25^^-^29

By far Schmidt's study,^19^ in 2010, is the most comprehensively descriptive study demonstrating the reliable anatomy (location and size) of the different IMAPs from the 1st one through the 5th, depicting the different sizes and suggesting the variable clinical applications for each IMAP flap.

When we compared the results of Schmidt et al^19^ with ours with respect to the maximally measured perforation distance from the sternum (being 2.5 cm in ours, and 4 cm in theirs) for the 4th IMAP—the previously well-proven to be the reliable and dominant supplying vessel to the medially based lower pole breast flap—we could conclude that if determined to raise this tissue flap as a pedicled one, it is better to avoid parasternal dissection closer than about 3 cm to the sternum to avoid jeopardizing the coming blood supply through these perforators. Moving to the 4th perforator maximum measured diameter in both studies, we will find that they were almost the same (2.1 mm in Schmidt's and only 0.1 mm less in our research), thus favoring the potential reliability of this vessel as a sufficient single pedicle for the flap, as was additionally evidenced by the aforementioned fluorescein injection test in our series.

Studying the 5th IMAP-relevant information comparatively between ours and Schmidt's was ignored as actually it would not have any impact on delineating the reliable vascular anatomy of the medially pedicled lower pole breast flap, because Schmidt's study already stated that the 5th perforator vascular territory lies below the inframammary fold, and so it is not included within the tissue flap in question. Therefore, including the 5th IMAP in the flap pedicle will not substantially influence the flap perfusion and furthermore will confine the rotational capacity of the flap.

Another difference between the results of the Schmidt et al study and this current study is the absence of two 4th IMAPs in the examined 10 breasts in the Schmidt et al study and presence of the 4th IMAPs in all the examined 20 breasts in this current study. Up to our knowledge, we consider our current study to be the only study—among the English literatures—that addressed exclusively the lower IMAPs, namely the 4th and 5th ones, regarding their size, location, potential number, and their supplying territory in the breasts of living women and not cadavers, and thus additionally proved the possibility of elevating the otherwise discarded inferior mammary dermoglandular tissue in reduction mammoplasties as a flap with known and reliable axial vascular pattern.

The singular case of lower breast tissue nonfluorescence (appeared dark blue) could be explained by failure of the dye to reach this dissected tissue part secondary to real-time perfusion compromise, which in turn, resulted from iatrogenic injury to the single, supplying 4th IMAP during parasternal dissection just before injecting the dye.

## CONCLUSION

Based on this study, the breast tissue below the areola that is typically excised in superior pedicle reductive mammary surgery can reliably be raised as an axial flap pedicled on both the 4th and 5th IMAPs or on the 4th perforator solely.

In our opinion, a preoperative color Duplex scanning of the lower IMAPs to ensure the presence at least of the 4th IMAP is highly advisable, or even a must in order to avoid flap vascular complications, as elevating the lower pole breast tissue as a medially based flap without at least visualizing the 4th interspace IMAP by color Duplex prior to surgery will not often be opportune.

## Figures and Tables

**Table 1:** Patients' ages, breast measurements, and individual IMAP diameters

Cases	Age, y	Suprasternal-NAC distance (right breast), cm	Right 4th IMAP diameter, mm	Right 5th IMAP diameter, mm	Suprasternal-NAC distance (left breast), cm	Left 4th IMAP diameter, mm	Left 5th IMAP diameter, mm
1	38	36	1	0.8	37	1.4	Undetectable
2	30	38	0.8	2.1	37	2	Undetectable
3	30	45	0.8	1	45	1	1.6
4	34	38	1.3	1	37	1.3	Undetectable
5	31	39	1.4	0.9	39	1.4	1
6	32	48	1.3	0.9	47	1.7	0.8
7	33	38	1.9	Undetectable	38	1.3	Undetectable
				0.6			0.5
8	34	35	1		35	1.2	
				0.7			0.5
9	28	37	1	0.9	38	0.7	1.1
10	42	38	1.1	0.7	39	1.1	0.9

IMAP indicates internal mammary artery perforator; NAC, nipple-areola complex.

**Table 2:** Comparison between both sides of examined breasts regarding suprasternal-to-NAC remoteness

	Breast side	Number	Minimum	Maximum	Mean	SD	P*
Suprasternal-NAC distance	Right	10	35 cm	48 cm	39.2 cm	4.07	1.00
	Left	10	35 cm	48 cm	39.2 cm	3.70	

NAC indicates nipple-areola complex; SD, standard deviation.

*The Student *t* test for equality of means.

**Table 3:** Data comparison between right-side IMAPs and their left-side counterparts regarding average diameters and remoteness from sternum

	Side	Number	Minimum	Maximum	Mean	SD	P*
Diameter, mm	Right	20	0.6	2.1	1.06	0.38	.509
	Left	17	0.5	2	1.14	0.4	
Distance from sternum, cm	Right	20	0.5	2.5	1.22	0.49	.368
	Left	17	0.5	2.5	1.05	0.6	

IMAP indicates internal mammary artery perforator; SD, standard deviation.

*The Student *t* test for equality of means.

**Table 4:** Data comparison between all the 4th IMAPs from both sides and the 5th IMAPs regarding their average diameters and remoteness from sternum

	Space	Number	Minimum	Maximum	Mean	SD	P*
Diameter, mm							
	4th	20	0.6	2	1.23	0.34	.021
	5th	17	0.5	2	1.14	0.4	
Distance from sternum, cm							
	4th	20	0.5	2.5	1.2	0.54	.546
	5th	17	0.5	2.5	1.08	0.56	

IMAP indicates internal mammary artery perforator; SD, standard deviation.

*The Student *t* test for equality of means.
